# Balance recovery and its link to vestibular agnosia in traumatic brain injury: a longitudinal behavioural and neuro-imaging study

**DOI:** 10.1007/s00415-024-12876-2

**Published:** 2025-01-15

**Authors:** Zaeem Hadi, Mohammad Mahmud, Elena Calzolari, Mariya Chepisheva, Karl A. Zimmerman, Vassilios Tahtis, Rebecca M. Smith, Heiko M. Rust, David J. Sharp, Barry M. Seemungal

**Affiliations:** 1 Centre for Vestibular Neurology (CVeN), Department of Brain Sciences, Charing Cross Hospital, Imperial College London, London, W6 8RF UK; 2 Department of Brain Sciences, Hammersmith Hospital, Imperial College London, London, W12 0NN UK; 3 Care Research & Technology Centre, UK Dementia Research Institute, Imperial College London, London, UK; 4 King’s College Hospital NHS Foundation Trust, London, SE5 9RS UK; 5 Department of Neurology, University Hospital Basel, Basel, Switzerland

**Keywords:** Vestibular recovery, Traumatic brain injury, Self-motion perception, Vestibular agnosia, Imbalance, Resting-state functional connectivity, Diffusion tensor imaging

## Abstract

**Background:**

Vestibular dysfunction causing imbalance affects c. 80% of acute hospitalized traumatic brain injury (TBI) cases. Poor balance recovery is linked to worse return-to-work rates and reduced longevity. We previously showed that white matter network disruption, particularly of right inferior longitudinal fasciculus, mediates the overlap between imbalance and impaired vestibular perception of self-motion (i.e., vestibular agnosia) in acute hospitalized TBI. However, there are no prior reports tracking the acute-longitudinal trajectory of objectively measured vestibular function for hospitalized TBI patients. We hypothesized that recovery of vestibular agnosia and imbalance is linked and mediated by overlapping brain networks.

**Methods:**

We screened 918 acute major trauma in-patients, assessed 146, recruited 39 acutely, and retested 34 at 6 months. Inclusion criteria were 18–65-year-old adults hospitalized for TBI with laboratory-confirmed preserved peripheral vestibular function. Benign paroxysmal positional vertigo and migraine were treated prior to testing. Vestibular agnosia was quantified by participants’ ability to perceive whole-body yaw plane rotations via an automated rotating-chair algorithm. Subjective symptoms of imbalance (via questionnaires) and objective imbalance (via posturography) were also assessed.

**Results:**

Acute vestibular agnosia predicted poor balance recovery at 6 months. Recovery of vestibular agnosia and linked imbalance was mediated by bihemispheric fronto-posterior cortical circuits. Recovery of subjective symptoms of imbalance and objective imbalance were not correlated.

**Conclusion:**

Vestibular agnosia mediates balance recovery post-TBI. The link between subjective dizziness and brain injury recovery, although important, is unclear. Therapeutic trials of vestibular recovery post-TBI should target enhancing bi-hemispheric connectivity and linked objective clinical measures (e.g., posturography).

**Supplementary Information:**

The online version contains supplementary material available at 10.1007/s00415-024-12876-2.

## Introduction

Traumatic brain injury (TBI) is the leading cause of chronic disability and death in young adults [1], and cause of death in 50% of over 65 year olds following a fall [2]. Vestibular dysfunction—typically with multiple central and peripheral (inner ear) diagnoses—causing dizziness and/or imbalance, affects c. 80% of acute TBI patients [3], and c. 50% at 5 years [4]. Importantly, persisting vestibular dysfunction post-TBI causes falls [5], and reduces return-to-work rates [5, 6] and long-term longevity [7]. Hence, understanding the mechanisms of vestibular dysfunction post-TBI is needed to develop targeted therapy for at-risk patients.

In contrast to subjective dizziness outcomes [8, 9], prospective objective vestibular data in TBI are sparse [10]. In TBI, damage to perceptual mechanisms uncouples symptoms from signs. Thus, objective vestibular activation with nystagmus may not evoke vertigo perception in patients—a syndrome called vestibular agnosia (VA) (see supplementary video), which is linked to falls [11], brain disconnection, and uncouples subjective symptoms and objective deficit [3, 12, 13]. This uncoupling of symptoms from signs was also previously noticed in TBI patients c. 90 years ago [14] by Glaser who noted in a series of 66 traumatic brain injury patients, “*Exceedingly difficult to understand, however, is the absence of true vertigo in head injuries, in spite of the presence of peripheral and central vestibular damage*”. Hence, tracking vestibular burden in TBI using subjective dizziness will be misleading. For example, BPPV (benign paroxysmal positional vertigo) prevalence amongst in-patient rehabilitation TBI cohorts was 7% [15] using screening via dizziness symptoms versus 58% [16] using systematic examination. In contrast, objective laboratory-measured balance in acute TBI was linked to both disrupted brain connectivity and laboratory measured VA [17], with the VA–imbalance overlap mediated by disrupted right inferior longitudinal fasciculus white-matter microstructure.

The key questions unresolved by our previous report [12] included the temporal pattern and the neuroanatomical correlates of recovery of imbalance, VA, and dizziness symptoms. We predicted that balance recovery would link to VA recovery behaviorally and via overlapping neuroimaging correlates. Hence, we conducted the first acute, prospective, longitudinal study assessing objective vestibular dysfunction in TBI patients who were highly characterised; clinically, behaviorally in the laboratory, and via structural and functional MRI. This report provides new longitudinal data relevant to recovery, in a cohort in which we previously reported only acute data [12, 18, 19].

## Methods

### Cohort recruitment and testing

Recruitment, consent (including patients lacking capacity), testing schedule (admission, 3 and 6 months), and pre-testing treatment of BPPV and residual migraine-phenotype headaches were previously reported [12].

Inclusions: (i) hospitalized patients with blunt head injury; (ii) 18–65 years old; (iii) preserved peripheral vestibular function. Exclusions: (i) active pre-morbid medical/neurological/psychiatric conditions; (ii) musculoskeletal condition impairing balance; (iii) substance abuse; (iv) pregnancy. Age–sex-matched healthy controls were tested once.

Ethical permission was provided by a local Research Ethics Committee (17/LO/0434). The principles of the Declaration of Helsinki were adhered to.

### Procedure (all repeated at 0, 3, 6 months)

Comprehensive testing (0, 3, 6 months) included evaluating peripheral and reflex vestibular function, vestibular perceptual testing, posturography, and neuroimaging (MRI at 0, 6 months).

### Vestibular perceptual thresholds

**Fig. 1:**
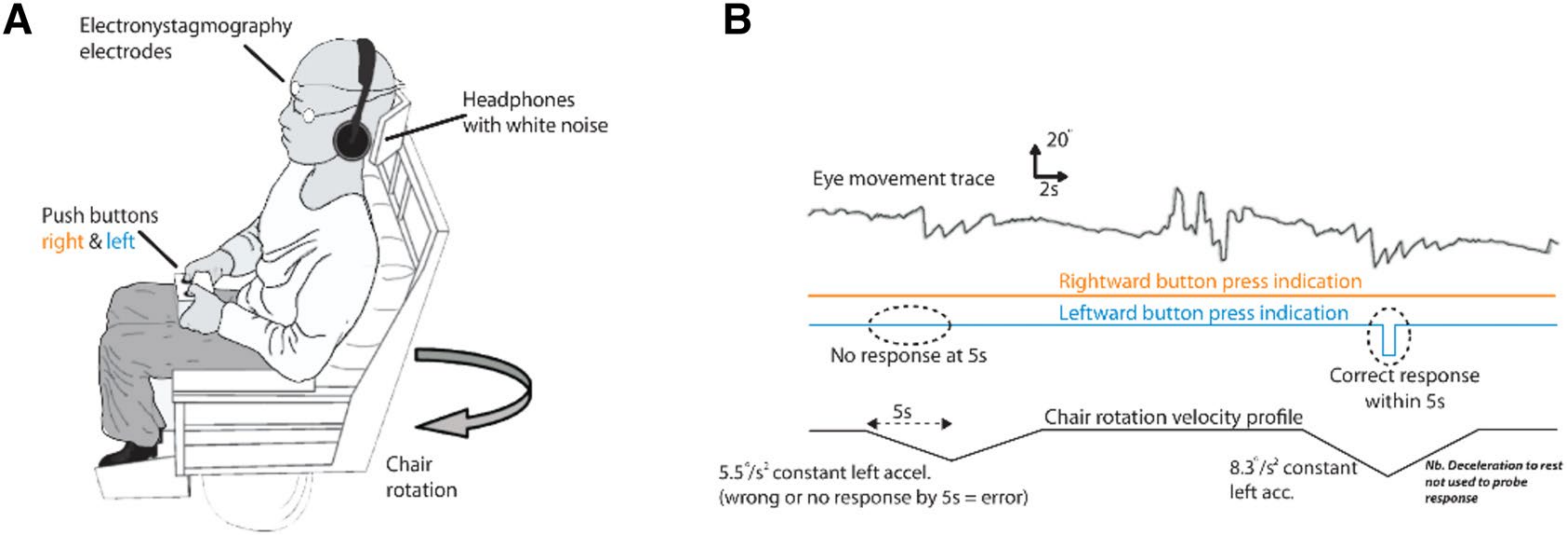
Vestibular agnosia measurement. **A** Patients were seated on a rotating chair in dark and rotated in yaw plane and were instructed to press right or left to indicate rightward or leftward rotation. Background noise was masked using white noise, whereas eye movements were also recorded. **B** Traces indicating eye movements, button-press by participant, and velocity profile of computer-controlled chair rotation

Vestibular perceptual thresholds (VPTs) of perceived self-motion during passive, yaw-plane, whole-body rotations in the dark, and the evoked vestibular ocular reflex (VOR) nystagmus were obtained [17]. Briefly, participants sat on a rotating chair in the dark with white-noise masking and were instructed to press a right or left button as soon as they perceived right- or leftward rotation (Fig. 1A, B).

### Posturography

We analysed root mean square (RMS) of sway measured via force platform, for condition "soft surface-eyes closed" (SC). In SC, balance is predominantly reliant upon vestibular cues as vision and proprioception are absent/degraded. Condition SC best discriminates TBI patients from controls [12].

### Clinical examination and questionnaires

Symptoms were assessed using the Dizziness Handicap Inventory (DHI) [20] and Activities-Specific Balance Confidence scale (ABC) [21]. Patient Health Questionnaire 9 (PHQ-9) [22] assessed depression severity. The patients were initially assessed on the ward by BMS and HMR; both experienced vestibular neurologists who performed a detailed and focused clinical examination including eye movement and vestibular neurological assessment including for signs of peripheral vestibular loss, e.g., doll's eyes and head impulse maneuver, assessing for nystagmus in the primary position with and without visual fixation, and via fundoscopy assessing for any nystagmus with and without visual fixation and assessing for peripheral (e.g. BPPV) and/or central positional nystagmus. Additionally, examination always included mental state and capacity, eye movements, Romberg, gait, tandem stance, tandem gait, limb tone, power and coordination, tendon and plantar reflexes, and sensory testing, including pain and joint position.

### Statistical analysis of objective and subjective vestibular measures

In the manuscript, the term objective vestibular function or objective recovery is used for the laboratory assessed measures of balance and VPTs, whereas the term subjective symptoms or subjective recovery is used for the subjectively acquired questionnaires (e.g., DHI and ABC).

We used R (https://www.R-project.org/, version 4.2.3; 2023-03-15) and JASP [23] (version 0.17.2) for statistical analyses and graphical outputs.

#### Link between recovery of balance and vestibular agnosia

To assess the mechanistic link between recovery from imbalance and vestibular agnosia, we performed three analyses, and hence, a corrected *P* < 0.0167 was considered statistically significant. The analyses included: i) a one-way repeated-measures ANOVA with factors group (VA+ and VA−) and timepoints (0 and 6 months) using sway RMS as outcome; ii) a correlation was performed between the absolute change in sway and absolute change in VPTs from acute to 6 month period; iii) two correlations were performed of absolute change in sway and absolute change in VPTs within VA+ and VA− groups as well. The Bartlett and Levene’s tests were used to assess for the equality of variances, whereas the normality of data distribution was assessed using the Shapiro–Wilk test.

As analyses with absolute changes also included patients who were at relatively normal levels, a separate logistic model was used to assess whether vestibular agnosia predicted composite vestibular recovery (balance and vestibular perception). Patients were categorized at 6 months as recovered/normal and non-recovered using the normative balance (mean: 36.37 and SD: 9.13) and vestibular perceptual thresholds (mean: 0.76°/s^2^; SD: 0.42) data from 37 healthy controls with a cut-off of mean + 2SD. This resulted in 12 patients (of 34 at follow-up) who did not make a full recovery to the normative levels. Patient recovery status was included in model as dependent variable, whereas the acute vestibular agnosia status (VA+ or VA) and sex (M/F) were added as factors. Since our cohort had more males than females (26 of 34 at follow-up), sex was added in the null model to control for its effects. The patients who recovered or had normal balance and vestibular perception acutely were coded as ‘1’, whereas patients who did not recover were coded as ‘0’ in our model.

#### Confirmatory analysis

To supplement the findings from the above logistic model, a post hoc analysis was performed to assess which of the group (VA+ or VA−﻿) was unbalanced at 6 months follow-up when compared to normative balance of healthy controls. Kruskal–Wallis rank sum test (due to unequal variances—Bartlett test) with post hoc Dunn test (Bonferroni adjusted) was used with sway RMS as outcome.

#### Link between subjective symptoms and objective vestibular function

To assess our prediction of lack of concordance between objective and subjective measures, six correlations between objective vestibular measures (balance and VPTs) and subjective measures (DHI, ABC, PHQ-9) were performed, and three correlations within subjective measures (DHI, ABC, and PHQ-9) were performed. Thus, a corrected p value for nine comparisons (*P* < 0.0055) was used for a correlation to be considered statistically significant. Shapiro–Wilk test was used to assess the normality assumption and Spearman’s rank correlation was performed if data were not normally distributed.

#### Link of subjective dizziness and imbalance with vestibular agnosia

To assess the longitudinal change of symptom scores (DHI) in different groups (VA+ and VA−), a one-way repeated-measures ANOVA with factors group (VA+ and VA−) and timepoints (0 and 6 months) was performed using DHI score as outcome.

## Neuroimaging

### Image acquisition

Structural and functional MRI images were acquired at time 0 and 6 months using a 3T Siemens Verio MRI scanner using a 32-channel head coil. The scanning protocol included: (i) 3D T1-weighted images acquired using MPRAGE sequence (image matrix: 256 × 256; voxel size: 1 × 1; Slices: 160; field of view: 256 × 256 mm; slice thickness: 1 mm; TR = 2300 ms; TE: 2.98 ms); (ii) T2*-weighted images sensitive to blood oxygenation level dependent (BOLD) signal for resting-state fMRI (image matrix: 64 × 64; voxel size: 3 × 3 × 3 mm^3^; slices: 35; field of view: 192 × 192 mm; flip angle: 80°; slice thickness: 3 mm; TR = 2000 ms; TE: 30 ms; volumes = 300; scan time = 10 min); (iii) DTI sequences were acquired in 64 directions with an isotropic voxel size of 2mm^3^, b = 1000 s/mm^2^ with four images with b = 0 s/mm^2^ (field of view = 256 × 256 mm; matrix size = 128 × 128; TR = 9500 ms; TE = 103 ms). Subjects were instructed to keep their eyes closed, stay awake, and avoid dwelling on any thoughts during resting-state imaging.

### Structural brain imaging: diffusion tensor imaging (DTI)

#### Pre-processing

Diffusion-weighted images were processed following the standard tract-based spatial statistics (TBSS) [24] pipeline in the FMRIB Software Library (version 5.0.8) [25], including correcting susceptibility and eddy current induced distortions and diffusion tensor fitting. Brain extraction was performed using HD-BET [26]. Tensor-based registration was performed using DTI-TK [27] and involved the creation of a group template using affine and non-linear diffeomorphic registrations followed by registration of participant diffusion imaging to the template. Images were warped to 1 mm isotropic space, and the mean FA map produced was thresholded at 0.2 to produce a white-matter skeleton. Subject FA data were then projected onto the mean FA skeleton. Each participant’s longitudinal change in FA was calculated with ‘fslmaths’ using the skeletonised images and the images were then merged into a single 4D image and used for group-level voxelwise analysis.

#### Group-level analysis

Normalized change values (z-scores) of both behavioural measures, vestibular perceptual thresholds (VPT) and the RMS sway in soft surface-eyes closed condition, were used as covariates to identify the interaction of recovery of vestibular perception and postural balance as well as their respective main effects in FSL [25].

#### Statistical analysis

All group level FA changes were evaluated using non-parametric permutation statistics. Threshold-free cluster enhancement (TFCE) [28] at *P* < 0.05 was used for multiple comparison correction. Findings are reported after correction for 6 contrasts at *P* < 0.0083, i.e., positive and negative correlation (equivalent to two one-sided t tests) for each main effect (change in sway & change in vestibular perceptual thresholds), and for the interaction of the same measures.

### Structural brain imaging: voxel-based morphometry (VBM)

#### Pre-processing

Data were pre-processed using the CAT12 Toolbox [29] using voxel-based morphometry (VBM) analysis [30] with SPM12 (http://www.fil.ion.ucl.ac.uk/spm/). The CAT12 automated preprocessing steps included spatial-adaptive non-local means denoising [31], and interpolation. Data were then bias corrected, affine-registered, and segmented using unified segmentation approach allowing skull-stripping, and the intensity normalized (to control for hyperintensities), and re-segmented using adaptive maximum a posteriori [32]. The segmentation was controlled for partial volume [33]. The data were transformed to MNI space and smoothed using a Gaussian kernel with FWHM of 6 mm [29].

#### Group-level analysis

For the group-level analysis, normalized change values of vestibular perceptual thresholds (VPT) and the RMS sway, were used as covariates to identify the interaction of recovery of vestibular perception and postural balance as well as their respective main effects in SPM. Total intracranial volume (TIV) was not included as a covariate since the analysis focuses on within subject changes for which TIV remains constant for all timepoints [29].

#### Statistical analysis

The GM images were analysed in group comparison using a flexible factorial design, which accounts for the longitudinal nature for each subject’s data [29]. Findings are reported after correcting for multiple comparisons at cluster level using family-wise error (FWE) correction at *P* < 0.05. Findings were also reported after correcting for the statistical evaluation of three contrasts, i.e., two main effects (vestibular perceptual thresholds and RMS Sway) and their interaction (corrected *P* = 0.016). Cluster height threshold for grey-matter specific VBM analysis was estimated to be F = 39.46 with an extent threshold of k = 10 voxels. The findings were shown using “BrainNet Viewer” [34], and in MNI coordinates.

## Results

### Recruitment and demographics

#### Participants

Recruitment and follow-up testing occurred between September 2017 and September 2020. N = 39 patients were recruited acutely. N = 34 completed behavioural testing at 6 months (one patient at 3 months and six at 12 months due to the COVID-19 pandemic). Numbers obtained for longitudinal imaging data were: VBM—33 patients, DTI—N = 27 patients (6 removed due to scanner change and 6 dropouts). 37 age- and sex-matched healthy controls (age: 40.78 ± 14.75 | Mean ± SD; 21 females) completed behavioural and neuroimaging testing once.

Resting-state imaging is only reported in supplementary due to a smaller sample size (N = 17; 6 removed due to scanner change, 6 dropouts; 10 removed following quality control).

### Demographics

Average age (Table 1) was 41.64 years (standard deviation: 13y), 28 M (71.8%); 11 F. The cause of injury was falls in 21/39; road traffic accident (RTA) in 15/39 (6/15 car passengers; 5/15 pedestrians, 4/15 not available). According to Mayo TBI severity criteria [35], 35/39 were moderate-to-severe and 4/39 mild-probable.

**Table 1:** Participants’ demographics and clinical details of injury, clinical examination, and reported history at each assessment

Subject	MOI	Severity MAYO	PTA	BPPV			Headache 0 month
				0 months	3 months	6 months	
1	RTA	Mod–Sev	1	+	−	−	−
2	RTA	Mod–Sev	1	+	+	+	+
3	Fall	Mod–Sev	1	+	−	NA	−
4	RTA	Mod–Sev	0	−	−	−	−
5	RTA	Mild-Prob	0	+	NA	NA	−
6	Fall	Mod–Sev	1	−	−	−	−
7	RTA	Mild-Prob	0	−	−	−	−
8	Assault	Mod–Sev	0	−	NA	NA	+
9	RTA	Mod–Sev	0	−	−	−	+
10	Fall	Mod–Sev	0	+	−	+	−
11	Fall	Mod–Sev	1	−	−	−	−
12	RTA	Mod–Sev	1	−	−	−	−
13	Fall	Mild-Prob	0	+	−	−	+
14	Fall	Mod–Sev	0	+	−	−	−
15	RTA	Mod–Sev	0	+	NA	+	−
16	Fall	Mod–Sev	0	−	NA	NA	−
17	Fall	Mod–Sev	0	+	−	−	−
18	RTA	Mod–Sev	0	−	−	−	+
19	Fall	Mod–Sev	0	+	NA	−	−
20	Fall	Mod–Sev	0	−	−	−	+
21	Fall	Mod–Sev	0	−	−	−	+
22	Fall	Mod–Sev	0	−	+	−	−
23	Fall	Mod–Sev	1	+	−	+	+
24	RTA	Mod–Sev	1	−	NA	+	−
25	Fall	Mod–Sev	1	+	NA	−	−
26	Fall	Mod–Sev	1	+	NA	NA	−
27	Assault	Mod–Sev	1	−	+	−	−
28	RTA	Mod–Sev	1	−	NA	NA	+
29	Fall	Mod–Sev	0	−	NA	−	−
30	Fall	Mod–Sev	0	+	−	−	−
31	Fall	Mod–Sev	0	+	+	+	−
32	RTA	Mod–Sev	1	−	−	−	−
33	Assault	Mod–Sev	1	−	NA	−	+
34	Fall	Mod–Sev	1	+	NA	−	+
35	Fall	Mild-Prob	0	+	−	−	+
36	RTA	Mod–Sev	1	+	NA	−	−
37	RTA	Mod–Sev	1	−	−	−	−
38	Fall	Mod–Sev	1	+	NA	−	−
39	RTA	Mod–Sev	1	−	NA	−	−

NA: not available; + /1: present; −/0: absent; MOI: mode of injury; Mod–Sev: moderate-to-severe; RTA: road traffic accident

### Clinical recovery of vestibular function

#### Resolution and recurrence of BPPV (benign paroxysmal positional vertigo)

Acutely, 19/39 (48.7%) had BPPV. All were treated by repositioning manoeuvres and were clear of BPPV at the time of objective vestibular function testing.

At 3-months, we assessed 24/39 patients, when 4/24 patients had BPPV (of whom 2 had ‘new onset’ BPPV); indicating a change in BPPV prevalence from 48.7% at time 0 months (19/39 patients) to 16.67% (4/24 patients) at 3 months.

At 6 months, we assessed 33/39 patients, where 6/33 had BPPV representing a 31.6% recurrence rate despite previous treatment to resolution. Any patient with BPPV at follow-up was treated until apparent resolution, returning one week later for testing.

Of the 146 patients, we examined acutely (screening), cases with skull fracture doubled the probability of having BPPV (vs. no skull fracture), indicating a force dependency of BPPV [19].

#### Assessment of peripheral and reflex vestibular function

Vestibular ocular reflex (VOR) thresholds of patients: 2.52°/s versus controls: 1.78°/s indicated normal reflex thresholds (t = 1.742, *P* > 0.05). Data of patients from video head impulse test (vHIT), caloric irrigation test, and electronystagmography (ENG) testing are reported in supplementary table (Table S1) and are also previously reported [12], all of which indicated that patients had preserved peripheral vestibular function.

#### Vestibular agnosia classification

Acutely, patients were categorized as having vestibular agnosia (VA+) or not (VA−) by comparison with healthy controls’ VPTs (mean: 0.76°/s^2^; SD: 0.42), using k-means clustering [18]. Patients with 1.99°/s^2^ or above acceleration threshold on either side (right- or left-sided rotations) were classified as vestibular agnosia [18]. 17 of 34 patients who completed follow-up testing had vestibular agnosia acutely (time 0).

#### Recovery of objective and subjective vestibular function

**Fig. 2:**
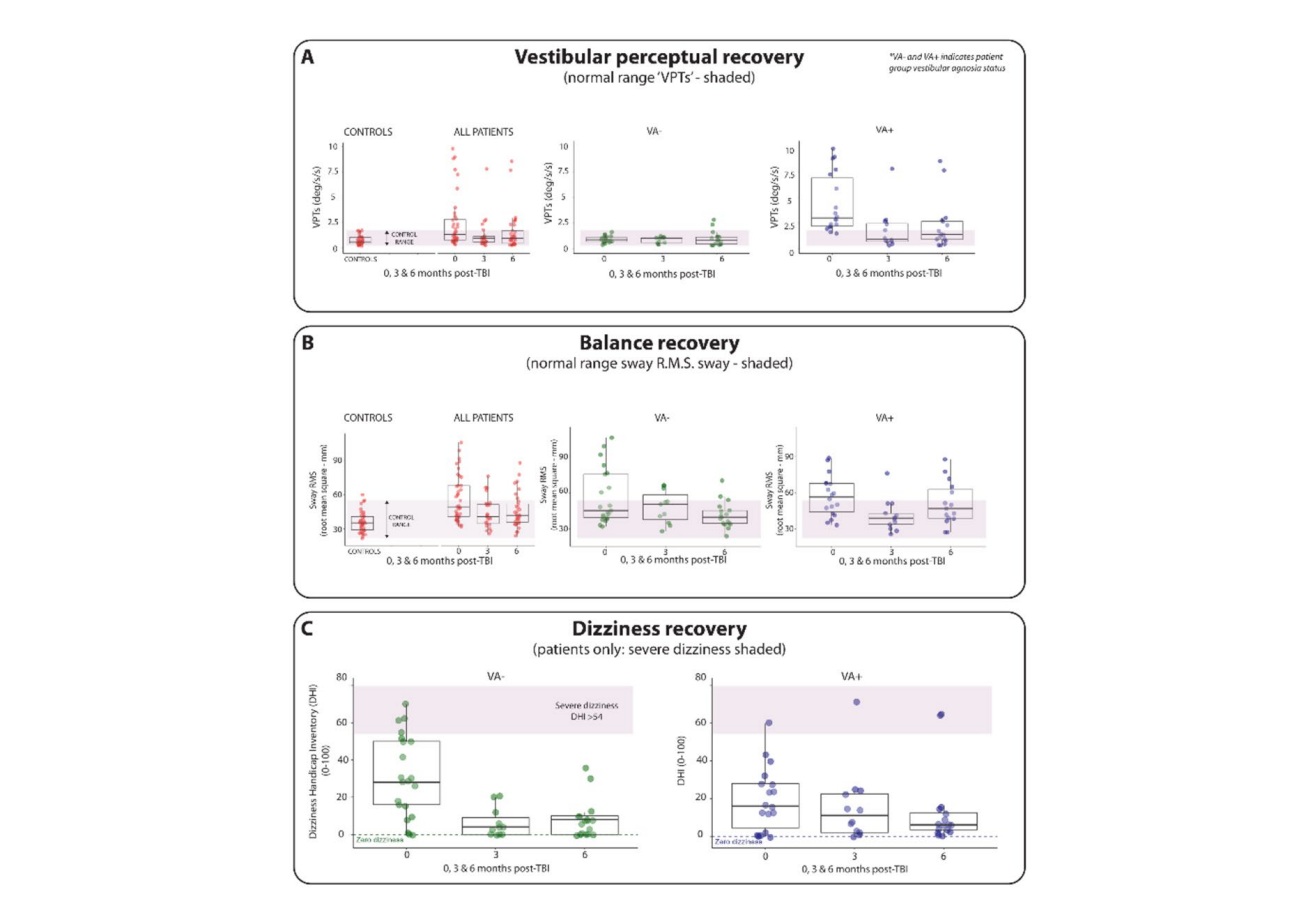
Recovery of dizziness symptoms, vestibular perceptual thresholds of self-motion and balance. **A** Recovery of objective vestibular perceptual function over time. Overall vestibular perceptual thresholds (VPTs) improved—i.e., reduced—over time across all patients (**A**—left panel, in red). For VA– patients, VPTs remained mainly within the normal range (**A**—middle panel, in green). For VA+ patients, there was persistence of abnormal VPTs at follow-up (**A**—right panel, in blue). **B** Recovery of objective balance function. Sway is shown as root mean square (RMS) of sway obtained during ‘eyes closed with soft surface’ condition. Overall sway improved—i.e., reduced—over time for all patients (B—left panel, in red). Balance recovery was worse, however, in the VA+ group with several patients showing persistently elevated sway RMS above the control range (**B**—right panel, in blue). **C** Recovery of dizziness over time. The dizziness handicap inventory (DHI) overall improved for both patients without vestibular agnosia (VA−; **C**—left panel, shown in green) and patients with vestibular agnosia (VA+; **C**—right panel, shown in blue)

**Fig. 3:**
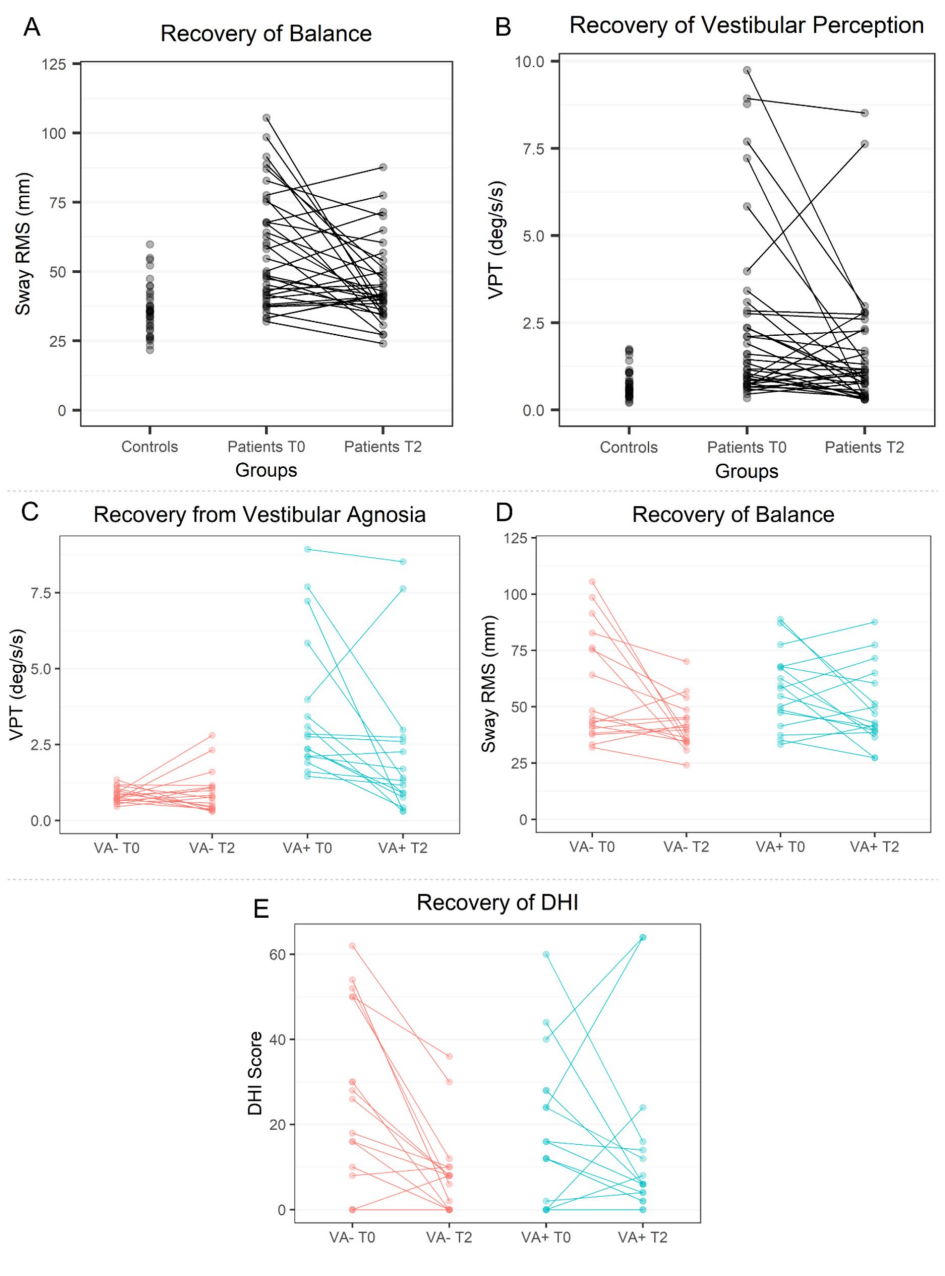
Longitudinal trajectories of recovery of balance, vestibular perceptual thresholds of self-motion, and dizziness symptoms. **A**, **B** Recovery of patients’ balance and vestibular perceptual thresholds compared to controls. Patients’ vestibular perceptual thresholds (VPTs) and balance were higher than controls acutely (T0 – 0 months) and generally reduced on 6-month follow-up (T2). **C **Recovery of vestibular perceptual thresholds. Overall VPTs improved from acute (T0—0 months) to follow-up (T2—6 months) in patients with VA+. Few patients with VA + got worse on 6-month follow-up (T2—6 months) (in blue), whereas a few VA− patients also developed VA (in red). **D** Recovery of objective balance function. Sway is shown as root mean square (RMS) of sway obtained during ‘eyes closed with soft surface’ condition. Overall sway improved—i.e., reduced—over time for VA– patients (in red). However, several VA =+ had persistent imbalance at 6-month follow-up (T2—6 months) and some also got worse (in blue). **E **Recovery of dizziness over time. Dizziness scores (via “Dizziness Handicap Inventory”) generally reduced for all patients except a few patients with VA + who got worse from acute (T0—0 months) to 6-month (T2—6 months) follow-up (in blue)

Raw data, means, and their distributions (via boxplots) are plotted in Fig. 2A–C for VPTs, balance, and DHI scores, whereas the trajectories of the longitudinal change of these measures for individual patients are plotted in Fig. 3A–E.

#### Patients’ balance recovery and its link to vestibular agnosia

In general, sway and VPTs improved (reduced) over time for all patients (Figs. 2A, B, 3A, B); however, balance recovery was worse in the VA+ group with several patients showing persistently elevated sway RMS or further worsening (increase) of sway (Fig. 3D). When we looked at the recovery of VPTs split into patients with acute VA+ vs. VA− (Fig. 2A and Fig. 3C), we noted that acute VA+ patients showed higher rates of persisting VA+ at 6–12 month follow-up. We also noted that two acute VA− patients developed vestibular agnosia at the 6-month follow-up (Fig. 3C), one of whom also developed imbalance despite having normal balance acutely.

Using the ‘eyes-closed-soft-surface’ balance condition as the outcome (which best discriminates imbalance for VA + vs. VA− vs. controls in acute TBI) [12], a within patient one-way repeated measures ANOVA with factors “Group” (VA+ vs. VA−) and “Time” (0 vs. 6 months) did not show a significant interaction F = 1.251 (*P* = 0.272, η_p_^2^ = 0.038).

**Fig. 4:**
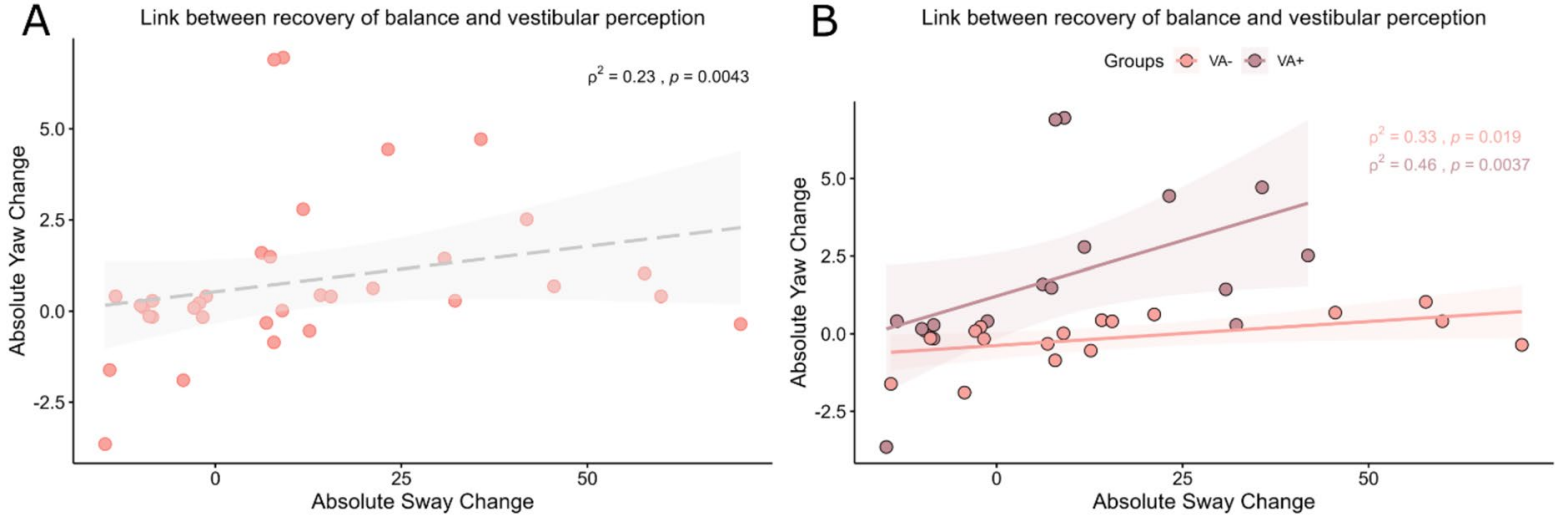
Relationship between recovery from vestibular agnosia and balance. **A** Correlation between continuous measures of vestibular perception and balance. Change in sway RMS and VPTs from acute to 6-month follow-up testing were linked. **B** Recovery of balance linked to VA recovery according to acute VA status. Longitudinal change in the balance-VA relationship appeared more robust in VA+ vs VA− patients (although the two correlations were not statistically different). (RMS: root mean square; VPTs: vestibular perceptual thresholds; VA+ : group with vestibular agnosia; VA−: group without vestibular agnosia)

Correlating the change of VPTs and change of sway from acute to follow-up (Fig. 4A) showed a positive correlation between change of VPTs and sway (ρ^2^ = 0.23, *P* = 0.0043; corrected *P* < 0.0167). Since correlation of all patients could mask the changes occurring at sub-group level, we then looked at the link between change of VPTs and change of sway from acute to follow-up by stratifying patients into VA + and VA− and performing separate correlations. Figure 4B shows that the correlation between balance and VPT change is more robust for the VA+ group (corrected *P* < 0.0167) than for the VA− group. However, the statistical difference between the two correlations is not significant (*P* > 0.05, 95% CI [−0.60 0.35]).

As the above analyses using absolute VPTs and sway changes included all patients, including those with relatively normal balance, we used a separate logistic model to assess if VA is a predictor of vestibular recovery (i.e., of balance and perception) in TBI (see Methods for classification details of recovered vs non-recovered patients). The model indicated that VA indeed is a marker of worse recovery of balance and vestibular perception (model summary: χ^2^(31) = 5.895, *P* = 0.015). Model coefficients are reported in Table 2.

**Table 2:** Logistic model predicting vestibular recovery

	Estimate	Standard error	Odds ratio	*z*	Wald statistic	df	*P*	Confidence interval
(Intercept)	0.702	0.835	2.017	0.840	3.937	1	0.047	[0.39 10.37]
VA (1)	−0.659	1.053	0.517	−0.626	0.392	1	0.531	[0.02 0.81]
Gender (1)	4.585	2.092	98.007	2.192	4.806	1	0.028	[0.57 27.34]

VA: vestibular agnosia. Confidence interval for odds ratio

To supplement the model’s conclusion, a confirmatory analysis was performed to assess the difference in sway between VA+, VA−, and healthy controls only at 6 month follow-up. A significant Kruskal–Wallis rank sum test (χ^2^ = 10.69; *P* = 0.0048) indicated a group difference. Post hoc analysis confirmed that VA+ patients had worse balance compared to controls (*P* = 0.0043), but not VA− patients (*P* > 0.05); however, VA+ vs. VA− was not statistically different when corrected (*P* > 0.05).

#### Recovery of subjective dizziness and perceived imbalance does not link to objective vestibular recovery

The DHI scores, used to assess subjective dizziness and balance, generally reduced at follow-up (Figs. 2C, 3E) except a few patients with vestibular agnosia (VA+) whose DHI scores increased (Fig. 3E). Notably, change in DHI did not correlate with change in vestibular perceptual thresholds (VPTs) or change in RMS sway (Table 3). Similarly, change in subjective balance measured via ABC scale, did not corelate with change in objectively assessed balance or with the VPTs (Table 3). Thus, subjective vestibular symptom scores did not correlate with their corresponding objective measures. In contrast, the change in subjective questionnaires (DHI, ABC, and PHQ-9) over follow-up were significantly correlated with each other (corrected for multiple comparisons; Table 3). It follows that symptomatic ‘dizziness’ cannot be used on its own as a proxy for vestibular recovery post-TBI.

**Table 3:** Correlations of subjective questionnaires and objective measures

Measure 1	Measure 2	Spearman’s rho	*P* value	95% Confidence interval
DHI	Balance^a^	0.135	0.447	[−0.209, 0.475]
ABC	Balance^a^	−0.234	0.191	[−0.573, 0.134]
PHQ-9	Balance^a^	0.347	^*^0.048	[−0.032, 0.628]
DHI	VPT	0.092	0.603	[−0.307, 0.450]
ABC	VPT	−0.298	0.093	[−0.605, 0.055]
PHQ-9	VPT	0.281	0.113	[−0.127, 0.595]
DHI	ABC	−0.507	^**^0.003	[−0.764, −0.157]
DHI	PHQ-9	0.732	^**^1.27 × 10^–6^	[0.450, 0.899]
ABC	PHQ-9	−0.550	^**^0.001	[−0.768, −0.245]

*DHI:* Dizziness Handicap inventory, *ABC﻿:* Activities-Specific Balance Confidence scale, *PHQ-9﻿:* Patient Health Questionnaire-9, *VPT﻿:* vestibular perceptual thresholds

*P < 0.05

**statistically significant at corrected p value for 9 correlations (*P* < 0.0055)

^a^Balance measured as root-mean-square sway

#### Link of subjective dizziness and imbalance with vestibular agnosia

Figures 2C and 3E show the difference in dizziness recovery when divided into patients with and without vestibular agnosia. Patients with VA (VA+) have lower acute dizziness mean score (Fig. 2C) despite having significantly worse acute clinical deficit [12]. While symptom scores for all VA− patients considerably improved at 6 months (Fig. 3E), symptom scores of some VA+ patients got much worse or did not improve (Fig. 3E).

An interaction of vestibular agnosia status (VA+ or VA−) and timepoints (acute and 6 month) with DHI scores as outcome indicated a borderline statistical difference in symptomatic recovery trajectories of VA subgroups (F(1,32) = 4.174, *P* = 0.049). A supplementary analysis further explores this non-linear link of VA with symptom scores (Supplementary Figs. S2 and S3).

### Neuroimaging correlates of vestibular recovery

#### Brain white matter microstructural (‘DTI’) connectivity and link to vestibular recovery

**Fig. 5:**
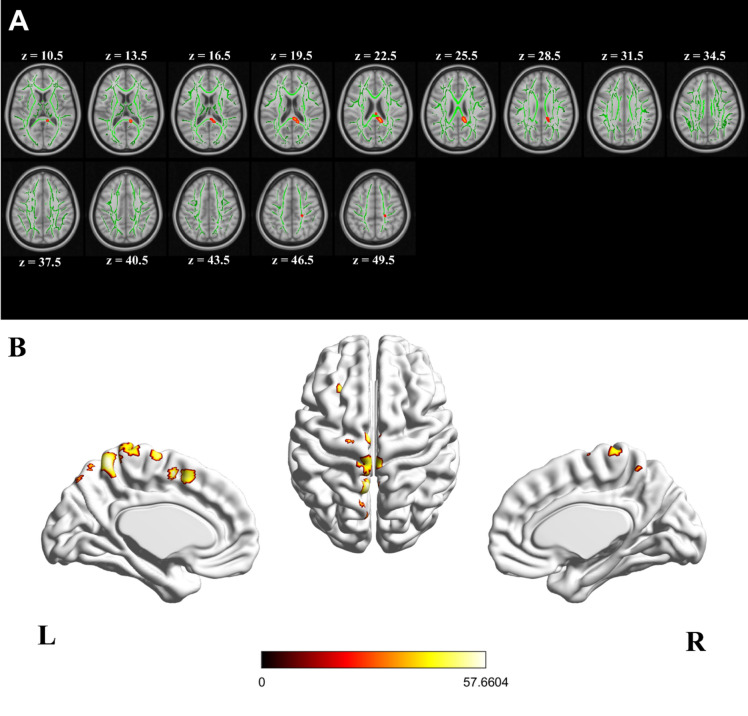
Interaction of change (Δ) in vestibular perceptual thresholds (VPTs), Δ sway, and the Δ connectivity values from different imaging modalities. L and R represent left and right hemisphere convention for all panels (**A, B**). **A** Diffusion tensor imaging analysis indicating a significant interaction (Δ VPT × Δ sway × Δ FA). Significant regions are highlighted in red and overlayed on mean FA skeleton (green) of all participants. (TFCE corrected findings at *P* < 0.0083). **B** Results from voxel-based morphometry (VBM) analysis showing interaction (Δ VPT × Δ sway × Δ Volume) at two clusters centred at left supplementary motor cortex, two at left precuneus, one at left precentral gyrus, one at left mid-frontal gyrus, and one at precentral lobule (FWE corrected at *P* < 0.05). (*Colour-bar indicate F statistic)*

A significant interaction of longitudinal change (Δ) of sway and of VPTs with longitudinal change (Δ) in fractional anisotropy (FA) was localised primarily in posterior corpus callosal regions including the splenium of the corpus callosum, forceps major, and body of the corpus callosum (TFCE corrected findings at *P* < 0.0083; Fig. 5A; Table 4). No main effects (of Δ sway or Δ VPTs) were found.

**Table 4:** Interaction and main effects of Δ VPT, Δ sway, and Δ GM volume over time

Cluster	Brain region	Cluster size	MNI x, y, z	Statistic value
Voxel-based morphometry analysis				
Interaction of Δ VPT, Δ sway, and Δ GM volume over time				
1	Left supplementary motor cortex	115	−4 3 54	F = 57.66^**^
2	Left precuneus	371	−4 −51 64	F = 56.11^**^
3	Left supplementary motor cortex, Left paracentral lobule, Right supplementary motor cortex	64	0 −9 72	F = 52.31^**^
4	Left precentral gyrus	10	−20 −10 74	F = 48.96^**^
5	Left middle frontal gyrus, Left superior frontal gyrus dorsolateral	20	−26 27 46	F = 48.36^**^
6	paracentral lobule, Right paracentral lobule, Left postcentral gyrus, Left precuneus	68	−4 −32 75	F = 45.56^**^
7	Left precuneus	11	−2 −76 52	F = 41.78^*^
Conditional main effect of Δ VPT				
1	Left supplementary motor area Left paracentral lobule Right supplementary motor area	82	0 −9 72	F = 56.16^**^
2	Right paracentral lobule Left paracentral lobule	24	24 2 −28	F = 46.25^**^
3	Left precentral gyrus	2	−20 −12 75	F = 42.18^**^
4	Left supplementary motor area	3	−3 3 52	F = 41.23^*^
Conditional main effect of Δ sway				
1	Left supplementary motor area Right supplementary motor area Left paracentral lobule	25	0 −10 74	F = 46.46^**^
Diffusion tensor imaging analysis				
Interaction of Δ VPT, Δ sway, and Δ FA values over time				
1	Splenium of corpus callosum^a^, forceps major^b^	215	11.6, −39.6, 21	−^†^
2	Right corticospinal tract^b^	12	22.3, −31.2, 47.6	−^†^
3	Body of corpus callosum^a^	5	2.6, −25.8, 22	−^†^

Δ: Change; FA: fractional anisotropy; − not available (as TFCE correction does not result in t values and associated degrees of freedom)

^*^FWE Corrected *P* < 0.05

^**^FWE corrected *P* < 0.0166 (corrected for 3 imaging contrasts/comparisons)

^†^TFCE corrected *P* < 0.0083 (corrected for 6 imaging contrasts/comparisons)

^a^ICBM-81 atlas [36, 37]

^b^JHU white-matter tractography atlas [38, 39]

#### Brain grey-matter volume correlates of behavioural change (balance and vestibular perceptual thresholds)

A significant interaction of the behavioural measures (Δ sway and Δ VPTs) with longitudinal change in grey-matter volume (Δ GM volume) was found primarily in the left hemisphere (Fig. 5B; FWE corrected *P* < 0.05), including two clusters in the left supplementary motor area, two in the left precuneus, and single clusters in the left mid-frontal gyrus, left precentral gyrus, and the paracentral lobule (Table 4; corrected for three comparisons *P* < 0.0166).

## Discussion

In this first acute-prospective-longitudinal study of vestibular function in acute hospitalised moderate-severe TBI patients, we report: (i) patients with acute vestibular agnosia (VA) display worse balance recovery than those without acute VA; (ii) VA-related balance recovery is mediated by (a) recovery of structural integrity of posterior corpus callosum; and (b) interhemispheric functional changes in the temporal occipital fusiform and the frontal pole cortical areas; (iii) subjective symptom scales do not predict objective vestibular recovery.

### Vestibular recovery: longitudinal change in imbalance, vestibular agnosia, and symptom scores

The recovery from imbalance and vestibular agnosia was linked such that acute VA status resulted in worse balance recovery at 6 months; several patients with vestibular agnosia either did not improve sway performance or worsened (Fig. 3D). While the occurrence of vestibular agnosia has previously been reported in several studies in elderly with imbalance [17, 40–44], none established the clinical relevance of vestibular agnosia for the prognosis of such individuals.

Notably, in the previous reports in elderly [41, 43, 44], the individuals reported having postural unsteadiness or postural instability; and while the authors argued that vestibular agnosia could result in lack of awareness about their unsteadiness, no objective findings were reported. Contrary to previous reports, we show a lack of concordance between objectively assessed balance and subjectively assessed balance using symptom scales indicating patients’ lack of awareness about their postural instability. This implies that subjective scores are a proxy for patients’ symptomatic well-being, but they do not inform upon objective recovery and hence poorly track brain injury-related recovery. A clear example of this uncoupling of subjective symptoms and objective signs is demonstrated in supplementary video where despite a clear reflex vestibular activation (i.e., observable nystagmus) via caloric irrigation, no vertigo perception is reported by a patient with vestibular agnosia. It follows that symptomatic ‘dizziness’ cannot be used on its own as a proxy for vestibular recovery post-TBI.

Some patients who did not have acute VA, developed VA at 6–12-month follow-up. Potentially, persistence or progressive VA worsening increases falls risk, either via undiagnosed vestibular conditions (e.g., BPPV) or worsening central postural control. Indeed, a recently published study confirmed that vestibular agnosia is a risk factor for postural instability and falls in multimorbid elderly [11]. Current studies prospectively tracking cognition and falls in TBI survivors should also consider tracking VA. Progression in VA could potentially mirror neurodegeneration and falls, both linked to long-term survival in TBI survivors [7].

### The brain mechanisms mediating recovery of vestibular function

Data linking vestibular perception and balance are sparse; however, the link was first theorised following the clinical observation of vestibular agnosia in an elderly patient with diffuse white matter small vessel disease, falls, and BPPV without vertigo [45]. We subsequently failed to find any effect of acute focal stroke upon vestibular perceptual thresholds [46], indicating that VA, and linked imbalance, was mediated by brain network dysfunction [47]. In the current study, we show for the first time the neuroimaging correlates of VA and linked balance recovery, refining our understanding of the brain networks supporting vestibular function and its recovery.

Longitudinal changes in white-matter microstructure (via DTI) of the splenium of the posterior corpus callosum were linked with the recovery of imbalance and vestibular agnosia. Indeed, interhemispheric disconnection in the genu, body, and splenium of corpus callosum was confirmed in unbalanced TBI patients compared to healthy controls [12]. More convincing however, is that TBI patients with imbalance had worse damage to the genu of the corpus callosum than TBI patients with normal balance [12]. These findings are congruent with that of our longitudinal fMRI data (supplementary Fig. S1, Table S3) and with our previous studies [12, 18], and buttresses the notion that interhemispheric disconnection in TBI leads to vestibular dysfunction with imbalance and vestibular agnosia.

Our longitudinal VBM findings link the recovery from imbalance and vestibular agnosia with volumetric change in left precuneus in the posterior parietal cortex (PPC), which has projections to an important vestibular processing region, the parieto-insular vestibular cortex (PIVC) [48], which previous studies have shown is transcallosally connected via the splenium [49]. That our VBM findings were primarily in posterior cortical regions again supports the notion that impaired vestibular perception and balance in acute TBI is an acute disconnection syndrome, and their recovery is correspondingly mediated by recovery of posterior interhemispheric connectivity.

Given that the vestibular system, more than any other sensory system, is tightly coupled to motor output circuits, it was not surprising that our structural analysis (DTI and VBM) also showed that vestibular recovery is also linked to recovery of primary and secondary motor cortical areas and their connections, including the corticospinal tract (Table 4), regions which have been shown in primates to receive and transmit vestibular signals as part of the vestibular cortical network [50]. Indeed, frontal cortical regions have been linked to short-term balance training [51] and long-term training in elite dancers [47]. Thus, damage to the extensive recurrent vestibular projections between primary and secondary motor areas [52], and other vestibular cortical hubs, explains how motor cortical damage impairs efferent control of balance in TBI [53].

## Limitations

Despite screening 1000 patients, given our strict inclusion/exclusion criteria, our data poorly generalize to elderly and multi-morbid cases, and those with peripheral vestibular injury which compounds central imbalance and expect to display worse imbalance and recovery. As a result, our data primarily reflect the impact of TBI, since we minimised confounds from premorbid brain, medical or psychiatric disease, and peripheral vestibular hypofunction. Our strict criteria reduced our final sample size; however, this still compares well with similar mechanistic albeit cross-sectional studies [47, 54]. In contrast, our prospective-follow-up improved the study power. Imaging the cerebellum and lower brainstem was affected by technical limitations (‘windowing’). Additional work is needed to more robustly assess the brainstem/cerebellar links to VA.

To measure vestibular symptom scores and perceived imbalance, we used “Dizziness Handicap Inventory” (DHI) scale; however, we acknowledge the limitations of this scale. A better scale is required for future studies in acute-TBI setting, which should be validated by its comparison with systematic neurological and objective assessments.

## Conclusion

In this first acute-prospective study assessing recovery of vestibular function, we show that the linked recovery of vestibular agnosia and imbalance is mediated by recovery of interhemispheric structural and functional connectivity. Patients with vestibular agnosia are thus at increased risk of falls due to: (i) damage to overlapping brain circuits that mediate balance and vestibular perception of self-motion; (ii) a lack of vertigo symptoms results in poor clinician recognition of treatable vestibular conditions. Given that subjective symptoms of dizziness unreliably indicate brain injury severity, its recovery, and any treatable vestibular diagnoses (e.g., BPPV), it follows that all patients with TBI require a detailed vestibular neurological assessment irrespective of symptoms. Our finding of progressive worsening of balance and vestibular perception in some individuals requires replicating in larger and more long-term follow-up studies, but it may represent progressive neurodegeneration. Longitudinal tracking of VA could represent a biomarker for detecting such deterioration as well as monitoring the effect of any treatment to ameliorate neurodegeneration-related imbalance. In any case, TBI survivors (like other vulnerable groups) [55] should be monitored for common treatable balance conditions, such as BPPV, irrespective of vertigo symptoms, and in so doing modify falls risk and potentially reduce long-term mortality from falls in this patient group.

## Supplementary Information

Below is the link to the electronic supplementary material.

Supplementary file1 (DOCX 1016 KB)

Supplementary file2 (MP4 5889 KB)

## Data Availability

Raw data that support the findings of this study are available from the principal investigator, upon reasonable request. The request would require a formal data sharing agreement, approval from the requesting researcher's local ethics committee, a formal project outline, and discussion regarding authorship on any research output from the shared data if applicable.
